# Socio-demographic predictors of food security among rural households in Langai district in Plateau-Nigeria: a cross-sectional study

**DOI:** 10.11604/pamj.2022.43.36.32704

**Published:** 2022-09-21

**Authors:** Philip Adewale Adeoye, Tolulope Olumide Afolaranmi, Antoinette Ngozi Ofili, Oluwabunmi Oluwayemisi Chirdan, Hadizah Abigail Agbo, Lydia Toluwanimi Adeoye, Tin Tin Su

**Affiliations:** 1 Department of Community Medicine, Jos University Teaching Hospital, Jos, Plateau State, Nigeria,; 2 Department of Community Medicine, University of Jos, Jos, Plateau State, Nigeria,; 3 Department of Community Health, College of Medical Sciences, University of Benin, Benin, Edo State, Nigeria,; 4 Knowledge Management Department, Nigeria Institute of Social and Economic Research, Ibadan, Oyo State, Nigeria,; 5 South-East Asia Community Observatory and Global Health, Jeffery Cheah School of Medicine and Health Sciences, Monash University Malaysia, Bandar Sunway, Malaysia

**Keywords:** Coping behaviours, empowerment, food security, household consumption, Nigeria security, women

## Abstract

**Introduction:**

household food insecurity is a critical social determinant of health globally. There is a rising global prevalence of food insecurity which affects households’ access to food of adequate quantity and quality. This study assessed the level of food security and its socio-demographic determinants among rural households.

**Methods:**

this is a cross-sectional community survey among 201 rural households in Langai district representing a total population of 1,284. Reported food security was assessed using Food Consumption Scores and Coping Strategy Index. Data were analysed with SPSS 21. Analysis was done to assess the level of food-security associated factors. Multivariable analysis was done to assess determinants of food security. P-value <0.05 is considered significant.

**Results:**

forty-three percent of all households have a total income greater than the national minimum wage. Overall, 21.4% are quality-food insecure (FCS), 6.5% have severe coping behaviour (CSI-food insecure) and 34.8% are highly economically vulnerable to food insecurity. Significant predictors of household food security include women earning than the basic monthly wage (AOR: 4.29 [95%CI: 1.34-13.71]; p=0.014); those without marital partners (AOR: 4.91 [95%CI: 1.01-23.90]; p=0.049); smaller household size (≤ 5) (AOR: 2.69 [95%CI: 1.16-6.24]; p=0.021); and those not receiving financial support (AOR: 2.68 [95%CI: 1.17-6.13]; p=0.020).

**Conclusion:**

almost a quarter of all households’ experience food insecurity with more than one-third being highly vulnerable to food insecurity. Efforts should be made to cushion the effect of global food insecurity among vulnerable populations and improve livelihood through improved agricultural practices to have sustainable and equitable food security.

## Introduction

Food security was defined by the UN to mean that all people, at all times, have physical and economic access to sufficient, safe and nutritious food to meet their dietary needs and food preferences for an active and healthy life [1]. Food security has four domains, namely: food availability, access, utilization and stability [2,3]. Food security ranges from the global, regional, national, local, household and individual levels of food insecurity. Globally, 821 million people are chronically hungry - with Africa having the highest prevalence (19.9%). The world´s food insecurity has been shown to have increased from 23.2% in 2014 to 26.4% in 2018 [3]. Households with food insecurity are uncertain about regular access to food-forcing them to make a compromise between food quantity and quality [3,4]. In Nigeria, the number of undernourished persons has increased from 10 million (2010) to 13 million (2016) [5]. These findings are particularly important as we attempt to achieve global zero hunger by 2030 [3].

Food insecurity is dependent on socio-economic, environmental, political and infrastructural factors in many countries of the world [4,6-11]. These factors have been associated with reduced purchasing power and access to food, reduced spending on basic needs and a shift from nutritious food to energy-dense food ensue. Food insecurity has been said to be an important predictor of malnutrition in many countries [3,4]. Due to the aforementioned state of food insecurity globally (and in Nigeria specifically), there is, therefore, the need to understand the socio-economic circumstances that underlie rural household vulnerability to food insecurity in North-Central Nigeria. The study assessed the prevalence of and socio-demographic factors that predict food security in Langai district of Plateau State, Nigeria.

## Methods

**Study design and settings:** a cross-sectional study. Plateau State is one of the North-central States of Nigeria; with a population of about 3.5 million. It is specifically known for its mountainous rock formations from which it derives its name; with its highest peak at 1,829 meters above sea level. It is mostly a rural State; with a cosmopolitan area around the State capital (Jos). The state is known for tin mining and agricultural production [12]. This study was carried out in Langai district over a period of 4 weeks in April 2019. Langai district is one of the 8 districts in Mangu Local Government Area, Plateau State, which is part of the Pyem chiefdom. It can be found at 9°37´0´´ North and 9°13´0´´ East. It is comprised of 3 communities, namely: Babban Rinji, Kadunun and Langai [13]. The predominant ethnic group is Pyem and the majority are of the Islamic faith.

**Selection and sample size determination:** a multistage sampling approach was used. Langai district was chosen due to an ongoing community diagnosis. There are 3 communities in Langai district. Langai community was chosen by simple random balloting. All households were subsequently selected for the study. Households surveyed were permanent residents of the communities while excluding households on transitory mobility (including nomads). Due to the complexity of African families, in this study, we define a household as a group of persons who eat, work together and share income and expenditures as a unit to ensure its welfare and production of food [11]. Households are the sampling units for the study and all necessary data were obtained from the mothers in the households. Where there was more than one mother in a household, a simple random sampling by ballot was done to select a mother. Mothers were selected because women have been said to be mostly at home during the day when study visits usually take place in most study settings; especially in developing countries. They are also in the best position to give realistic information about food access and quality [14]. Advocacy to households was done while doing house numbering of the various households in Langai. Two community guides assisted with advocacy activities. Respondents were encouraged to freely participate and to opt-out of the study at any stage of the study. They were informed about the purpose and objectives of the study and probable outcomes. They were assured that the information gathered will solely be used for educational purposes. Concerns and questions were subsequently addressed. Minimum sample size was calculated using the Cochran formula [15]:


$$n=\frac{Z^{2}pq}{d^{2}}$$

Z=standard normal deviate at 95% confidence level =1.96; p=proportion of food security in a prior study=0.863 [16]; q=proportion of alternate outcome (1-p) =0.137; d=error at 5%. Calculated sample size was 181. Calculating 10% non-response, sample size = 199 households.

**Data collection:** data collection was interviewer-administered and the questionnaire was semi-structured. It was divided into socio-demographic of respondents, Food consumption scores, socio-demographic characteristics of households, food acquisition and storage, community factors affecting food access, the practice of agriculture and the Coping Strategy Index (CSI). Total household income is the income of all household members (≥15 years) who are engaged in income-generating activities. Educational status is basic if at least Junior High [17]. Food security was measured in this study by exploring household food consumption, coping strategies and share of expenditure on food. Household (HH) food consumption is meant to reflect consumption practice (including food diversity) in the last 7 days. The gold-standard for measuring food security are household per capita kilocalories and individual anthropometric measurements. However, it should be known that nutritional status is a multidimensional matrix and food security is one among many. Their use is often affected by logistic, feasibility and applicability issues, and the fact is that they do not capture essential elements of food security [18,19]. Rapid tools have been subsequently developed which can be used for emergency assessments, short-term assessments, valid and applicable to fieldwork [2,18].

Food security data was collected through the adoption of two validated tools for measuring food security: Food Consumption Scores (FCS) and Coping Strategy Index (CSI) [2,18,20-22]. Food Consumption Scores (FCS) have been said to measure dietary diversity, food frequency and quality, and to some extent quantity. It was developed by the World Food Programme (WFP) in 1996 and has extensive validation in sub-Saharan Africa, South America, the Caribbean and Asia [19-21]. It is a proxy indicator for caloric intake and household dietary quality [18-22]. Coping strategy index was developed to measure locally adapted coping strategies/behaviours that individuals and families engage in during shortfall in food consumption. It was adapted by the World Food Programme. It measures food, quantity, acceptability, economic access and, to a lesser extent, quality [2,18,20-22]. It is meant to identify households with food insecurity, the causes and impact of food insecurity, monitoring and impact evaluation [20,21]. It has a well-grounded construct and is significantly well correlated with other measures of food security. The share of food expenditure on food was measured by determining the proportionate spending on food of the total household income [20,21]. It is an indication of food insecurity vulnerability. Irrespective of the current food security status, a decline in income would likely be associated with a reduction in food quantity and quality of food consumed. Thus, the higher the household´s proportionate spending on food; the greater the probability of poor food access. It also reflects the proportionate share of total expenditure on food; and correlates with FCS and CSI [2,20].

A comprehensive demonstration was made to data collectors on procedures for collecting data from respondents. This was done by role-playing and pre-testing activities. Training on the ethics and protocol of the study was done by specialist public health physicians of the Department of Community Medicine, University of Jos, Nigeria. Data collection was carried out by 30 research assistants who are proficient Hausa language speakers; as Hausa language is the most commonly spoken language in this community. The questionnaires were translated into Hausa language and back-translated before the commencement of the survey. Before the data collection, landmarks were noted and the research team was divided into groups for data collection. Each group started from the central market and moved towards a pre-identified landmark closest to the boundary of the community; and returned to the market through the adjacent street. This movement was repeated by the various units until all households were reached. The first house was the closest house to the interviewer since all households were intended to be covered.

**Data processing and analysis:** data analysis was done by SPSS. Descriptive statistics were presented in median, interquartile range, frequency, proportions and charts. Charts were constructed using Excel 2010. Food consumption scores are calculated by aggregating frequency (f) of consumption of country-specific foods into eight standard food groups with food-specific weights (w), which depend on their relative nutritional value (quality). The weighted product summation of the food groups gives the FCS composite score. The higher the composite score the better the level of household food consumption; and thus, food security. This can be categorised into thresholds of poor (0-21), borderline (21.5-35) and acceptable (>35) to describe the state of food security [2,18-22]. Food consumption scores were further reclassified into food secure (acceptable category) and food insecure (poor + borderline categories) [18].

The CSI was obtained by aggregating coping strategies with severity weights. The higher the aggregate score, the higher the coping behaviours reported, and thus, the more food insecure [2,20,21]. The weighted product summation of the coping strategies gives the CSI composite score. This was subsequently categorized into low CSI (0-50); medium (51-100) and high (> 100). Low CSI = food secure; medium CSI = mild food insecurity; high CSI = moderate-severe food insecurity [23]. Coping strategy index was reclassified into food secure and food insecure (mild + moderate-severe food insecurity). [18]. The food insecure category signifies severe coping behaviour.

Percentage income expenditure on food was subsequently categorized into low (<50%), medium (50-60%), high (65-75%) and very high (>75%) vulnerabilities to food insecurity. Household spending on food greater than 75% of income is adjudged vulnerable to food deprivation [20]. It was reclassified as lower vulnerability (low + medium + high expenditures) and higher vulnerability (very high expenditure). As recommended by Maxwell D *et al*., cross-classification between FCS and CSI was done to improve the measurement of food insecurity [18]. We hypothesized that socio-demographic characteristics of households, food acquisition and storage, community factors affecting food access, the practice of agriculture and the Coping Strategy Index (CSI) are influencers of food security; and thus, independent variables. The outcome variable is food security (using FCS). Chi-square was employed in determining the relationship between categorical variables and food security; except when the conditions for chi-square are not satisfied. To identify the predictors of food security, variables with a probability value of P-value < 0.25, were entered into the omnibus multivariable logistic regression model [24]. A p-value < 0.25 was chosen because a relaxed p-value will not allow us to miss any important variable that may predict food security; as may be seen with a tighter p-value. A p-value < 0.05 is statistically significant. The predictive power of the model (78.6%) is high, indicating that food security is being significantly explained by the independent variables. It also shows a non-significant Hosmer-Lemeshow.

**Ethical consideration:** approval for this study was obtained from the Plateau State Ministry of Health Ethics Review Committee (MOH/MIS/202/VOL.T/X). Study participation was voluntary and written informed consent was obtained before participants were included in the study.

## Results

**Socio-demographic characteristics of households:** a total of 201 households participated in the study; representing a total population of 1,284 with an average of 6 persons per household. About 70% of respondents were between the ages of 25 and 64; of which more than half (53.7%) of all households are of the predominant tribe. More than two-thirds (62.7%) of all households are of the Islamic faith; with 70.1% without a basic education; and 89.6% married; with 67.7% of households engaging in non-farming occupations. Most (93.0%) household heads are males. Almost two-thirds (56.7%) of all households have respondents (women) who earn less than the monthly minimum wage. About half (52.7%) of all household heads earn less than the monthly minimum wage. Almost two-thirds (56.7%) of all households have a total income of at least the minimum wage. More than two-thirds (62.7%) of all households source their income from both farm and non-farm sources. More than two-thirds (64.7%) of all households receive financial support from family members. Most (87.6%) of all house ownerships are family-owned. About two-thirds (62.7%) of all households have more than six household members (Table 1).

**Table 1:** socio-demographic characteristics of households in Langai district (n=201)

Variables	Frequency (n)	Proportion (%)
**Age**		
<25 years	48	23.9
25-64 years	142	70.6
≥ 65 years	11	5.5
**Age (years) median [IQR^ǂ^]**	35 (27-49)	
**Tribe**		
Pyem	108	53.7
Non-pyem	93	46.3
**Religion**		
Islam	126	62.7
Christianity	75	37.3
Education*		
Nil basic education	141	70.1
At least basic education	60	29.9
**Marital status**		
Living with partner	180	89.6
Living alone*	21	10.4
**Occupation**		
Non-farming*	136	67.7
farming	65	32.2
**Monthly income (women)** ^$^		
≥N18,000 (≥US$ 50.07)	87	43.3
<N18,000 (<US$ 50.07)	114	56.7
**Household head**		
Male	187	93.0
Female	14	7.0
**Monthly income of household heads** ^$^		
<N18,000 (<US$ 50.07)	106	52.7
≥N18,000 (≥US$ 50.07)	95	47.3
**Total Monthly household income** ^$^		
<N18,000 (<US$ 50.07)	87	43.3
≥N18,000 (≥US$ 50.07)	114	56.7
**Main source of income of household**		
Non-farm only	29	14.4
Farm only	47	23.4
Both	125	62.2
**Family member contribution to family income**		
Yes	130	64.7
No	71	35.3
**House ownership**		
Family-owned	176	87.6
rented	25	12.4
**Number of persons in households**		
≤ 5	75	37.3
≥ 6	126	62.7
**Number of persons in households median [IQR^ǂ^]**	6 (5.0-8.0)	

* Educational status is basic if at least Junior secondary School; ǂ=Interquartile range; living alone include single/divorced/widowed; nNon-farming=housewife, trading among others; $= exchange rate at $1= 359.47 as at April19, 2019.

**Level of food security among households:** of all households 21.4% are food insecure: this represents quality and quantity food insecurity (diversity and frequency of food consumption); with 34.8% highly economically vulnerable to food insecurity and 6.5% having severe coping behaviour- a measure of food insecurity. Cross-classification of FCS and CSI shows that 10.5% of all households are significantly insecure in at least one dimension of food security; while 2% are food insecure in quality and quantity (X^2^=9.702; p=0.025) (Table 2).

**Table 2:** level of food security among households in Langai district (n=201)

Variables	Frequency (%)
FCS* category	
Food insecure	43 (21.4)
Food secure	158 (78.6)
**Average FCS median (IQR)**	52 (40.0-69.0)
%* income on food acquisition	
Lower vulnerability	131 (65.2)
Higher vulnerability	70 (34.8)
**Average % income spent on food**	70 (30.0-80.0)
CSI* category	
Food insecure (severe coping behaviours)	13 (6.5)
Food secure	188 (93.5)
**Average CSI median (IQR)**	15 (2.0-35.0)

* FCS=Food Consumption Score; IQR: Interquartile Range; %=percentage; CSI=Coping Strategy index

**Figure 1:**
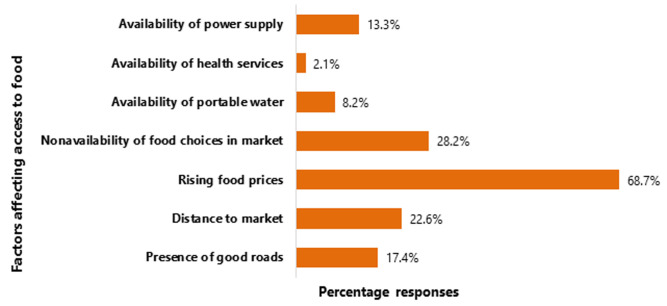
factors affecting access to food in Langai district; source: data analysis of fieldwork

**Factors affecting access to food:** Figure 1 shows that rising food prices are the most important factor (68.70%) affecting access to food in Langai district. Other top concerns of households about access to food are the non-availability of food choices in the market and distance to the market.

**Household socio-demographic characteristics associated food security:** Table 3 shows that a higher proportion of the predominant population (pyem), most households with women respondents earning more than the minimum wage, most households whose total household monthly income is more than the minimum wage and most households who receive no family financial support are significantly food secure. A higher proportion of households with smaller family sizes are significantly food secure.

**Table 3:** association between socio-demographic characteristics of households with food security in Langai district (n=201)

Variables	Food insecurity frequency (%)	Food security frequency (%)	X2	P-value
**Age (years)**			0.812 L	0.717L
<25	12 (25.0)	36 (75.0)		
25-64	28 (19.7)	114 (80.3)		
≥ 65	3 (27.3)	8 (72.7)		
**Tribe**			4.435	0.035*
Pyem	17 (15.7)	91 (84.3)		
Non-pyem	26 (28.0)	67 (72.0)		
**Education status**				
Nil basic education	28 (19.9)	113 (80.1)	0.662	0.416
At least basic education	15 (25.0)	45 (75.0)		
**Marital status**			-	0.259f
Living with partner	41 (22.8)	139 (77.2)		
Living alone^ǂ^	2 (9.5)	19 (90.5)		
**Occupation (major)**			0.593	0.441
Non-farming^ǂ^	27 (19.9)	109 (80.1)		
Farming	16 (24.6)	49 (75.4)		
**Monthly income (women)** ^$^			8.938	0.003*
≥N18,000(≥US$ 50.07)	10 (11.5)	77 (88.5)		
<N18,000(<US$ 50.07)	33 (28.9)	81 (71.1)		
**Household head**			-	0.738f
Female	2 (14.3)	12 (85.7)		
Male	41 (21.9)	146 (78.1)		
**Monthly income household head** ^$^			3.825	0.050
<N18,000(<US$ 50.07)	17 (16.0)	89 (84.0)		
≥N 18,000(≥US$ 50.07)	26 (27.4)	69 (72.6)		
**Total household income** ^$^			4.918	0.027*
<N18,000(<US$ 50.07)	25 (28.7)	62 (71.3)		
≥N18,000(≥US$ 50.07)	18 (15.8)	96 (84.2)		
**Members' contribution**			4.960	0.026*
Yes	34 (26.2)	96 (73.8)		
No	9 (12.7)	62 (87.3)		
**Source of household income**			3.065L	0.222L
Non-farm only	4 (13.8)	25 (86.2)		
Farm only	14 (29.8)	33 (70.2)		
Both	25 (20.0)	100 (80.0)		
**House ownership**			0.115	0.734
Family	37 (21.0)	139 (79.0)		
Rented	6 (24.0)	19 (76.0)		
**Number of persons in households**			4.622	0.032*
≤ 5	10 (13.3)	65 (86.7)		
≥ 6	33 (26.2)	93 (73.8)		

ǂ=Educational status is basic if at least Junior High; living alone include single/divorced/widowed; non-farming=housewife, trading among others; *significance at p<0.05; f=Fisher's Exact Test; L=Likelihood Ratio; $= exchange rate at $1= 359.47 as at April 19, 2019.

**Socio-demographic predictors of food security among households:** significant predictors, after excluding variables with a p-value of at least 25%, are expressed in binary logistic regression. Households with women earning at least the minimum monthly wage are 4.29 times more likely to be food secure compared to those households who earn less (AOR: 4.29 [95%CI: 1.34-13.71]; p=0.014). Households that receive no familial financial support are 2.71 times more likely to be food secure compared to those who receive familial financial support (AOR: 2.71 [95%CI: 1.14 - 6.40]; p=0.023). Those living alone (without a partner) are almost 5 times more likely to be food secure compared to those living with partners (mostly married) (AOR: 4.91 [95%CI: 1.01-23.90]; p=0.049). Household sizes of at most 5 (≤5) is 2.69 times more likely to be food secure compared to those households with at least 6 occupants. (AOR: 2.69 (1.16-6.24); p=0.021) (Table 4).

**Table 4:** socio-demographic predictors of food security among households in Langai district

	Bivariate logistic regression		Multivariate model	
Variable	OR^ǂ^(95%CI)	P-value	AOR^ǂ^(95%CI)	P-value
**Age**		0.626	**------**	**------**
Younger	1.41(0.35-5.55)			
Elderly (ref)	1			
**Tribe**		0.037*^§^		0.165
Pyem	2.08(1.04-4.13)		1.70 (0.80-3.60)	
Non-pyem (ref)	1		1	
**Religion**			**------**	**-------**
Islam	1.44(0.73-2.86)	0.295		
Christianity (ref)	1			
**Education status**		0.417	**------**	**-------**
Nil basic education	1.35(0.66-2.75)			
At least basic education (ref)	1			
**Marital status**		0.178^§^		0.049*
Living alone	2.80(0.63- 2.53)		**4.91(1.01-23.90)**	
Living with partner (ref)	1		**1**	
**Occupation status**		0.442	**------**	**-------**
Non-farming	1.32(0.65-2.67)			
Farming (ref)	1			
**Monthly income (women)** ^$^		0.004*^§^		0.014*
≥ 18,000(≥US$ 359.47)	3.14(1.45-6.80)		**4.29 (1.34-13.71)**	
< 18,000(<US$ 359.47) (ref)	1		**1**	
**Household head**		0.506	**------**	**-------**
Female	1.69(0.36-7.83)			
Male (ref)	1			
**Monthly income of household head** ^$^				
< 18,000(<US$ 50.07)	1.97(0.99-3.92)	0.053^§^	2.83(0.47-16.83)	0.254
≥ 18,000(≥US$ 50.07) (ref)	1		1	
**Total household income** ^$^				
≥ 18,000(≥US$ 50.07)	2.15(1.08-4.27)	0.028*^§^	2.61 (0.48-14.15)	0.267
< 18,000 (<US$ 50.07) (ref)	1		1	
**Members' contribution**				
No	2.44(1.10-5.44)	0.029*^§^	**2.71 (1.14-6.40)**	0.023*
Yes (ref)	1		**1**	
**Source of household income**			**------**	**-------**
Non-farm only	1.83(0.60-5.58)	0.287		
At least a farm source (ref)	1			
**Number of persons in households**				
≤ 5	2.31 (1.06-5.01)	0.035*^§^	**2.69 (1.16-6.24)**	0.021*
≥ 6 (ref)	1		**1**	
*Significance at p<0.05				

§= significance at p<0.250 which were included in the multivariable models; ǂ=Educational status is basic if at least Junior High; living alone include single/divorced/widowed; non-farming = housewife, trading among others; OR/AOR = Odd Ration/Adjusted OR; $= exchange rate at $1= 359.47 as at April 19, 2019.

## Discussion

This study determined food security levels and predictors of household food security. Almost a quarter of all households were food insecure and more than three-quarters were food secure. Food insecurity was significantly higher than the cross-classification food insecurity of 10.5% among households-which is not frequently used in defining household food security in the literature despite its inherent benefits in preventing over- and under-estimation of food insecurity [18]. The level of food insecurity is similar to studies done in rural Pakistan and some regions of Canada [6,7,10]. Food insecurity is however significantly lower in the study area compared to those reported in Niger, regions of Ethiopia; Northern Cameroon, Zanzibar and Yemen [9,11,25-27]. Food insecurity in the study area was higher compared to rural Cambodia [16]. This variation might be due to climatic and frequency of insurgency factors that might have affected food production activities. The period of the study (April) is the planting season when households that depend on their farm products for food have finished their stocks and mostly depend on market sources.

This study showed a higher proportion of households with very high income expenditure on food compared to households in Nepal [28]. This will pose a higher risk of vulnerability to food insecurity during times of economic shocks. Higher expenditures were however seen in farming regions of Tanzania, Nicaragua and South Africa [28,29]. The poorer a household the larger the share of their budget on food consumption (Engel´s law) [30]; and the lower the spending on housing, utilities, durable goods, education and other basic needs [28].

The greatest concern affecting food availability expressed by households is the increasing food prices - which are related to lack of financial access according to this study. This corroborates a 2019 Food Agriculture Organisation (FAO) global report on food insecurity, observations in rural Pakistan, Niger and South Africa where increasing food prices, poverty and lower-income were stated as factors affecting food security [3,6,7,11,29,31]. The rate of the slow but steady rise in inflation has continually reduced the purchasing power of most households. Increasing desertification, epidemics and economic hardships have compounded the cost of most food items in most countries. Women's, and not men's, higher income is a strong predictor of food security although household income plays a huge role in ensuring food security according to this study. This is similar to studies in Tanzania, where improved women's income leads to improved family food security [8]. It has been said that improved women´s income leads to improved financial security, autonomy, health and well-being [8,32,33]. Increasing income for men is usually spent on capital projects and extra-household activities. Most income increase for women is spent on household needs; of which food is a priority. Unfortunately, most women are hindered by a lack of control over economic assets, poor decision-making opportunities and limited financial power to drive household food security in many low and middle-income countries.

This study showed that a higher proportion of households with no financial support from family members have proportionally higher food consumption levels compared to those with financial support. This is similar to studies in Niger, South Africa and Canada where in-kind payments or aids and social assistance have been said to be a hindrance to long-term food security. This has been said to lead to the inability of households to negotiate the quality and quantity of support received and a greater likelihood of being vulnerable to a lack of other basic daily needs [10,11,29,34].

Study findings showed that living in a non-partnered household (single, divorced and widowed) increases the likelihood of food security compared to those in partnered (married) households. This may be due to limited liability to feeding more adults in households, especially during the planting season when the study was done. There may also be a lesser financial burden on these households; especially when individuals are gainfully employed. This differs from observation from a Canadian study where single parentage, unattached and couples with children have a higher likelihood of food insecurity compared with couples without children [10]. This may also be due to cultural differences between western cultures and the cultures of the developing world. Vulnerable and less westernised individuals often report having received and given support compared to fully-westernized individuals [35]. Smaller households are more likely to be food secure compared to larger households according to this study. This is similar to studies from east Africa, Lebanon, Niger, Kenya, Uganda, Tanzania, Ethiopia, India and rural Pakistan. This is because large family size negatively impacts food calorie availability, consumption and production [7,9,11,34,36].

Limitations observed in this study included the lack of universally acceptable thresholds for food security - hence the need to use multiple measures for food security. The use of multiple measures affords this study comparability with FAO-WFP surveys. Also, the use of cross-classification prevents the over-estimation of food insecurity [18]. But for comparability with other studies, both single food security measures and cross-classification are presented in this study. Some of the questions may be culturally sensitive and answers are subjective which may not be congruent with real food insecurity and coping behaviours exhibited by households. This was mitigated by assuring respondents that the study is tied to relevant intervention that will ameliorate medium- and long-term household food insecurity. The time of the data collection was short to minimize recall bias which this study could have been at risk of.

This study measured a one-time household experience of food security compared with time series. The cross-sectional nature of this study will not allow the population to be studied over time. Further studies will enrich our knowledge by studying the seasonal trend of household food security. Other studies could also explore the impact of climate change and the COVID-19 pandemic on food security in various settings. This study may have limited generalizability because of its sample size; it nevertheless agrees with other studies and provides an idea about the state of food security in rural communities.

## Conclusion

This study showed that almost a quarter of households were food insecure. More than one-third of all households spent more than three-quarters of the household budget on food. Strong predictors of food security in these rural households include higher women income, non-partnered (unmarried) households, non-receipt of familial support and smaller household sizes. As a result of the level of food insecurity, efforts should be geared toward the initiation and implementation of policies and programmes that empower families; specifically targeted at the girl-child and women in general. There should be an increased emphasis on small family sizes through family planning. Rural families and communities may be empowered by improving livelihood to improve agricultural productivity; especially vulnerable families to ensure the sustainability of short-term benefit for a long-term gain of any aid given. Policies should be in place to ensure the stability of food prices since it could be deduced from the results that an increase in prices is one of the factors leading to food insecurity. Thus, there is a need to incorporate innovative, comprehensive, socially sensitive, gender-sensitive and sustainable measures towards addressing food insecurity and vulnerability among families and communities experiencing food insecurity. This will reduce food insecurity; improve agricultural productivity and empowerment among communities.

### What is known about this topic

- Household food security is an important social determinant of health;
- There is a rising prevalence of household food insecurity, globally, due to various factors;
- Rural households in conflict regions are particularly vulnerable to food insecurity.

### What this study adds

- Socio-economic factors are significant predictors of food security;
- This study provides evidence of food insecurity among rural populations and the need to improve the livelihood of vulnerable populations.
